# Overcoming doramectin failure: field efficacy of pour-on 5% fluralaner and an experimental 1% oral formulation for the prevention of *Cochliomyia hominivorax*–associated umbilical myiasis in newborn calves

**DOI:** 10.1186/s13071-026-07455-5

**Published:** 2026-06-17

**Authors:** Murilo Damasceno Brunet de Freitas, Daniel de Castro Rodrigues, Pablo Eduardo Martins de Paiva, Siddartha Torres, Fernando de Almeida Borges

**Affiliations:** 1 Parasitic Diseases Laboratory, Faculty of Veterinary Medicine and Animal Science, Federal University of Mato Grosso do Sul, Av. Senador Filinto Müller, 2443, Campo Grande, MS Brazil; 2 MSD Animal Health, São Paulo, SP Brazil; 3 Merck Animal Health, 2 Giralda Farms, Madison, NJ 07940 USA

**Keywords:** New World screwworm, Cattle, Avermectins, Isoxazolines

## Abstract

**Background:**

Myiasis caused by *Cochliomyia hominivorax* larvae is a reemerging challenge in the Americas due to the recent reappearance of this species in areas where it had been eradicated and reports of doramectin inefficacy, despite its recognition as the most effective avermectin for prevention in cattle. As an alternative, 5% fluralaner administered via pour-on has shown 100% efficacy in the treatment and prevention of myiasis. Therefore, this study aimed to evaluate the efficacy of three antiparasitic treatments, namely 1% doramectin administered subcutaneously, 5% fluralaner pour-on, and 1% fluralaner administered orally, in preventing umbilical myiasis caused by *C. hominivorax* in newborn calves.

**Methods:**

The study was conducted on a commercial beef cow–calf farm in Brazil. On day 0, during umbilical care, 200 newborn calves were randomly allocated to 4 groups: negative untreated control; 1% doramectin subcutaneously (200 µg/kg); 5% fluralaner pour-on (2.5 mg/kg); and 1% fluralaner orally (0.5 mg/kg, experimental formulation). All umbilical stumps were disinfected with 10% iodine tincture (10–30 s) without larvicidal compounds. Umbilical sites were evaluated on days 3, 7, and 14 for the presence of *C. hominivorax* larvae and lesion scores (0–3).

**Results:**

The results showed marked differences among treatments in the prevention of umbilical myiasis. In the control group, myiasis occurrence was 26% (95% CI 15–39%), confirming the high field risk of the disease. Treatment with 1% doramectin did not reduce (*P* = 0.8153) myiasis occurrence (22%; 95% CI 12.7–35.2%), showing a relative risk reduction of only 15.4%. Pour-on 5% fluralaner reduced myiasis occurrence to 4% (95% CI 1.1–13.4%), corresponding to a preventive efficacy of 84.6%. Despite proven 100% fluralaner efficacy, maternal licking may have contributed to the removal of the pour-on product in newborn calves, potentially reducing skin residues and being associated with the occurrence of umbilical myiasis. Orally administered 1% fluralaner showed 100% preventive efficacy. Umbilical lesion scores corroborated these findings, with a predominance of score 0 in fluralaner-treated groups.

**Conclusions:**

Doramectin was ineffective in preventing umbilical myiasis in newborn calves, whereas orally administered fluralaner showed 100% preventive efficacy. Pour-on fluralaner did not achieve complete preventive efficacy, possibly due to maternal licking at the product application site in calves.

**Graphical Abstract:**

**Figure:**
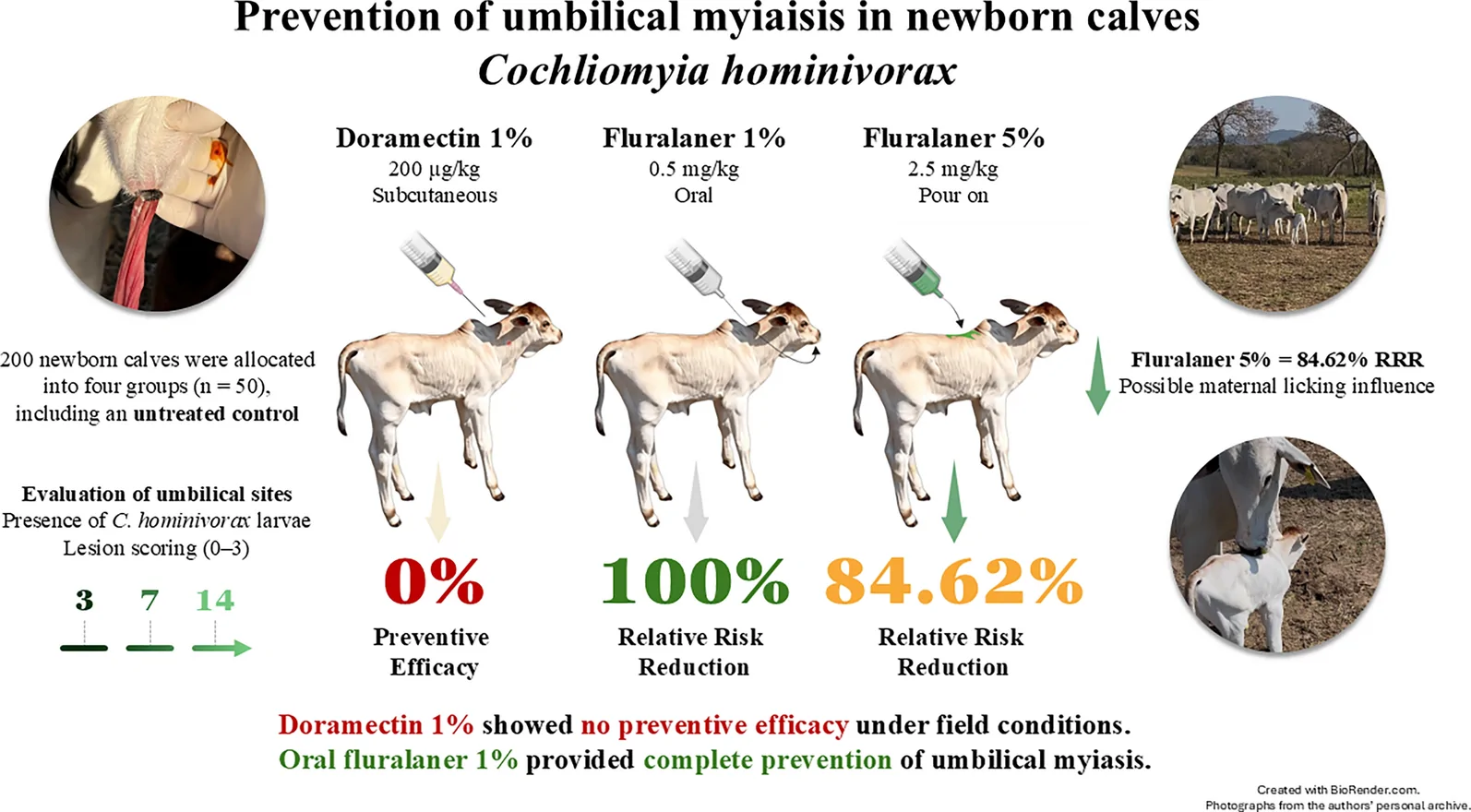

**Supplementary Information:**

The online version contains supplementary material available at 10.1186/s13071-026-07455-5.

## Background

*Cochliomyia hominivorax*, the New World screwworm (NWS) fly, is an obligate parasitic dipteran whose larvae invade living tissues of warm-blooded animals, causing traumatic myiasis associated with substantial animal health and economic losses. Because of its epidemiological relevance and transboundary impact, myiasis due to *C. hominivorax* remains a priority target for surveillance and eradication initiatives in the Americas [1].

In the current scenario, this species poses renewed challenges due to its reemergence in parts of Central and North America [2]; reports of reduced preventive efficacy of doramectin [3], the avermectin most commonly used for chemoprophylaxis; and the high susceptibility of newborn calves, particularly to umbilical infestation [4]. Together, these factors highlight the need to investigate alternative chemical strategies to improve prevention and control under field conditions.

The eradication of the NWS in the USA and Mexico was accomplished through a binational sterile insect release program conducted between 1972 and 1991, later extended to Central America and reaching Panama in 2001, at an estimated cost of US$955 million (1960–1991) [2]. Nevertheless, within just 4 years, new cases of myiasis caused by *C. hominivorax* reemerged, spreading from Panama (2021) to Mexico (2025). Given the imminent risk of reintroduction into North America, Valdez-Espinoza et al. [2] recommended control strategies on the basis of four main pillars, including the treatment of infected animals, underscoring the urgency of clinical studies evaluating new therapeutic alternatives.

Newborn calves represent the most susceptible category for the prevention of NWS myiasis in Brazil. Additional risk factors include routine management practices that may result in skin injuries, as well as high tick burdens that impair skin integrity [5]. The long-acting macrocyclic lactones have greatly facilitated myiasis prevention, making their administration the most widely adopted control strategy [3, 4]. Among the avermectins, doramectin has demonstrated the highest preventive efficacy in cattle [6, 7] and has been routinely administered in Brazil for more than three decades, particularly at birth [7, 10].

Recent reports highlight two major limitations of doramectin for the prevention of umbilical myiasis in newborn calves: reduced efficacy and potential toxicity. Preventive failure of macrocyclic lactones against umbilical myiasis has been recently documented in South America [3, 8]. In addition to efficacy issues, safety concerns must also be considered. Although doramectin is generally regarded as clinically safe, including for young animals, two outbreaks of doramectin intoxication in newborn calves have been described following accidental overdose during prophylactic use in Brazil [9].

In 2014, a new chemical class with strong ectoparasiticidal activity in dogs and cats was developed [10]. Subsequently, in response to the growing scenario of ectoparasite resistance in cattle, a pour-on formulation for this species was developed and registered for use in Brazil in 2022, demonstrating 100% efficacy for both the treatment and prevention of myiasis in cattle following administration of 5% fluralaner pour-on [4]. In December 2025, the U.S. Food and Drug Administration (FDA) conditionally approved this formulation for the prevention and treatment of New World screwworm (NWS) myiasis, as well as for the treatment and control of cattle fever tick (*Rhipicephalus microplus*) in beef cattle 2 months of age and older and in replacement dairy heifers less than 20 months of age.

In this study, we evaluated an experimental oral fluralaner formulation. This is justified by practical, welfare, and pharmacotechnical considerations associated with currently available treatments. Subcutaneous doramectin, although widely used, may cause injection-site pain, raising animal welfare concerns in the immediate postnatal period, and under field conditions, inadequate practices such as the reuse of syringes and needles may increase the risk of complications. Likewise, fluralaner topical (pour-on) formulations can be influenced by environmental factors, as rainfall occurring shortly after application may hypothetically reduce drug absorption and systemic bioavailability. Therefore, an oral formulation may represent a noninvasive alternative that minimizes handling-related stress, avoids injection-associated risks, and is less susceptible to environmental interference, potentially providing more predictable systemic exposure in newborn calves.

The aim of this study was to evaluate the efficacy of doramectin and fluralaner, administered orally and as a pour-on formulation, in the prevention of umbilical myiasis caused by *C. hominivorax* in newborn calves.

## Methods

### Study site

The experiment was conducted on a commercial beef cattle farm located in Porto Murtinho, Mato Grosso do Sul State, Midwest Brazil. The farm had a documented history of umbilical myiasis, despite routine preventive treatment of calves with doramectin at birth. The region has a tropical semi-humid climate (Köppen–Geiger Am), with a hot, rainy summer and a dry winter, mean annual temperature of 25.5 °C, and average annual precipitation of approximately 1432 mm.

The facilities complied with basic welfare and environmental management standards appropriate for the animal category evaluated in this study. Throughout the experimental period, cattle were maintained under the same routine farm management and environmental conditions. The study was conducted in accordance with Good Clinical Practice (VICH guideline GL9).

### Experimental animals

To ensure homogeneous experimental groups, Nelore calves and industrial crossbred calves were selected. Most animals were born from fixed-time artificial insemination (FTAI), while a smaller proportion originated from natural mating (clean-up bulls). All calves were newborns, with body weights ranging from 30 to 40 kg, clinically healthy, and in good nutritional condition. Each animal was individually identified with a numbered ear tag.

Calves’ dams were initially kept in maternity paddocks and monitored daily to allow prompt calving assistance when necessary. Animals were maintained on established pasture and had ad libitum access to mineral supplementation and fresh water. Due to the large number of experimental animals, it was not feasible to maintain all calves in a single paddock. Therefore, animals were evenly distributed among treatment groups to ensure a similar proportion of each treatment within each paddock.

Umbilical care was performed by immersion of the navel stump in 10% iodine tincture for 10–30 s, without the application of any larvicidal compound. Excessively long umbilical cords were trimmed using sterile scissors approximately two finger-widths (≈3 cm) from the abdominal wall, followed by immersion in 10% iodine solution.

### Experimental design

The study was conducted as a randomized, negative-controlled efficacy trial. At the time of umbilical care (designated as day 0, D0), 200 calves were allocated into 4 groups according to a randomized block design. All groups were balanced regarding sex and breed composition, including male and female Nelore and crossbred calves (Table 1). Treatments were randomly assigned within blocks of four animals as follows: group (1) 1% fluralaner (experimental formulation provided by MSD Animal Health), administered orally at 1 mL/20 kg body weight (0.5 mg/kg), dose defined by the manufacturer in previous clinical studies; group (2) 5% fluralaner (Exzolt^®^ 5%, MSD Animal Health), administered topically (pour-on) at 1 mL/20 kg body weight (2.5 mg/kg); group (3) 1% doramectin (Dectomax^®^, Zoetis Animal Health), administered subcutaneously at 1 mL/50 kg body weight (200 µg/kg); and group (4) untreated control.

**Table 1:** Distribution of animals according to sex and breed composition in each group

Group	Number of animals	Sex		Breed	
		Male	Female	Nelore	Crossbreed
1	50	29	21	32	18
2	50	30	20	33	17
3	50	31	19	34	16
4	50	28	22	34	16

Because the inclusion of 200 animals required the use of calves born on different dates, day 0 (D0) was defined individually for each animal as the day on which umbilical care and preventive treatment against myiasis were performed.

### Treatments

To simulate field conditions, in which most farms do not weigh calves at birth, thereby preventing accurate calculation of antiparasitic dosage, a fixed-dose regimen was adopted. Calves received 1 mL of 1% doramectin, 2 mL of 1% fluralaner administered orally, or 2 mL of 5% fluralaner administered topically (pour-on formulation), according to their respective treatment groups.

Although a fixed-dose approach was used, body weight was estimated using a bovine weight tape to ensure that all treated animals received doses within the label-recommended range for the commercial products or within the predetermined dose established for the experimental formulation.

Animals that developed active myiasis at any evaluation timepoint received rescue treatment consisting of an organophosphate-based larvicidal spray (Matabicheira FortDodge Aerosol^®^, Zoetis Animal Health), combined with antibiotic and antiinflammatory therapy (Terramicina^®^ Plus, Zoetis Animal Health), and 2 mL of 1% fluralaner administered orally. Following rescue treatment, these animals were immediately withdrawn from the study.

### Umbilical evaluation

Umbilical assessments for the presence of myiasis and lesion scoring were performed on days 0, 3, 7, and 14. As previously described, animals from any group that developed myiasis during the posttreatment period received rescue therapy and were subsequently withdrawn from the study.

To evaluate the preventive efficacy of the formulations, the umbilical stumps of calves were carefully examined for the presence of *C. hominivorax* larvae, regardless of larval instar. Lesions were classified as active when at least one live *C. hominivorax* larva was detected.

Umbilical lesion scores were assigned according to the following criteria (Rodrigues et al., 2010):

Grade 0: Umbilical structures undergoing normal involution, without pain or inflammatory changes;

Grade 1: Local inflammation characterized by pain on palpation, increased local temperature, and/or thickening and induration of the region;

Grade 2: Inflammatory process associated with the presence of purulent discharge;

Grade 3: Similar to grade 2, accompanied by rectal temperature above 40 °C.

## Statistical analysis

The proportions of animals presenting myiasis infestation in the control and treated groups were used to estimate the risk in each group. The relative risk (RR) was calculated as the ratio between the risk observed in the treated group (Rt) and that observed in the control group (Rc), according to the equation:

RR=Rt/Rc

Relative risk reduction (RRR) was estimated from the relative risk using the formula:

RRR=1-RR

Absolute risk reduction (ARR) was calculated as the difference between the risks observed in the control and treated groups:

ARR=Rc-Rt

Because sample sizes were similar across groups, ARR corresponded to the percentage efficacy calculated using the formula:

$$Efficacy(\% ) = [1 - (T/C)] \times 100$$

as recommended by Brazilian Ministry of Agriculture Ordinance No. 48/1997 (MAPA).

In this study, the effect of the evaluated treatments was expressed as preventive efficacy, also referred to as relative risk reduction. Because sample sizes were equivalent across experimental groups, both measures yielded numerically identical estimates and reflected the same clinical and epidemiological relevance.

Additionally, the number needed to treat (NNT) was estimated as the inverse of the absolute risk reduction:

NNT=1/ARR

The odds ratio (OR) was calculated using the formula OR = (a×d)/(b×c) on the basis of a 2 × 2 contingency table. The 95% confidence interval (95% CI) for the OR was estimated using the Baptista–Pike method.

The presence or absence of *C. hominivorax* larvae in the umbilical stump was analyzed using Fisher’s exact test (two-tailed), with a significance level of 5%. Statistical analyses were performed using GraphPad Prism version 10.6.1 for Windows (GraphPad Software, San Diego, CA, USA).

## Results

The absolute and cumulative numbers of umbilical myiasis cases in treated and untreated calves naturally exposed to *C. hominivorax* are presented in Table 2.

**Table 2:** Cases of myiasis and cumulative number of newborn calves with active umbilical myiasis on each posttreatment day (PTD) in treated and untreated groups naturally exposed to *Cochliomyia hominivorax*

Group	Cases of myiasis (cumulative number of calves with active umbilical myiasis in parentheses)/PTD					
	3		7		14	
Control	4		6	(10)	3	(13)
1% doramectin	5		4	(9)	2	(11)
5% fluralaner	2		0	(2)	0	(2)
1% fluralaner	0		0	(0)	0	(0)

Marked differences were observed among treatments regarding the prevention of umbilical myiasis. The proportion of affected animals in the untreated control group (26%; 95% CI 15–39%) confirms the substantial risk of disease occurrence under field conditions in the absence of preventive measures (Table 3).

**Table 3:** Risk estimates and preventive efficacy measures against umbilical myiasis in newborn calves treated with doramectin or fluralaner under natural exposure to *Cochliomyia hominivorax*

	Control	1% doramectin	5% fluralaner	1% fluralaner
Proportion (%)	26	22	4	0
95% CI	15–39	12.7–35.2	1.1–13.4	0.0–7.1
RR (%)	–	84.62	15.38	0
95% CI	–	41.9–100	3.6–64.6	
RRR (%)	–	15.38	84.62	100
95% CI	–	0–58.1	35.3–96.3	
ARR	–	4	22	26
95% CI	–	0–20.4	8.1–35-8	13.6–39.55
NNT	–	25	5	4
Odds ratio	–	0.8028	0.118	0
95% CI	–	0.31–2.01	0.025–0.55	
*P*-value*	–	0.8153	0.0038	0.0001

*RR* relative risk, *RRR* relative risk reduction (analogous to preventive efficacy), *ARR* absolute risk reduction, *NNT* number needed to treat

*Fisher’s exact test (two-tailed)

Treatment with 1% doramectin did not result in a significant reduction in the occurrence of umbilical myiasis compared with the control group. The proportion of affected animals remained similar (22%; 95% CI 12.7–35.2%), with a limited preventive efficacy (15.38%; 95% CI 0–58.1%) and an odds ratio close to unity (OR 0.80; 95% CI 0.31–2.01%), a finding corroborated by Fisher’s exact test (*P* = 0.8153). These results indicate that, under the evaluated conditions, the product did not meet the expected efficacy criteria for the prevention of neonatal umbilical myiasis.

In contrast, treatment with 5% fluralaner, administered as a pour-on formulation, showed a marked preventive effect, reducing the proportion of affected calves to 4% (95% CI 1.1–13.4%). The preventive efficacy was 84.62% (95% CI 35.3–96.3%), accompanied by an absolute risk reduction of 22 percentage points (95% CI 8.1–35.8) and a number needed to treat (NNT) of 5, meaning that five calves must be treated to prevent one case of myiasis. The odds ratio significantly below unity (OR 0.12; 95% CI 0.025–0.55), together with a *P*-value of 0.0038, confirms a statistically significant association between treatment and reduced odds of umbilical myiasis occurrence (Table 3).

The experimental 1% fluralaner formulation showed the highest preventive effect among the evaluated protocols, with no cases recorded during the observation period. Risk estimates indicated complete relative risk reduction and strong statistical significance, supporting its epidemiological and operational relevance under natural challenge conditions (Table 3).

Umbilical lesion scores corroborated the findings regarding myiasis occurrence among the evaluated groups. The control group and the group treated with 1% doramectin showed a higher frequency of mild-to-moderate lesions, consistent with the greater incidence of myiasis observed in these groups. In contrast, the groups treated with fluralaner, either orally or as a pour-on formulation, showed a predominance of score 0, indicating preservation of umbilical integrity. In the 5% fluralaner (pour-on) group, the two animals with score 1 corresponded to the only recorded cases of myiasis, whereas in the group treated with 1% fluralaner (oral administration), no lesions or cases of myiasis were observed, further supporting the preventive effect of the treatment (Table 4).

**Table 4:** Distribution of calves by umbilical lesion score across treatment groups

Group	Umbilical lesion scores/number of calves			
	0	1	2	3
Control	37	12	0	1
1% doramectin	39	11	0	0
5% fluralaner	48	2	0	0
1% fluralaner	50	0	0	0

## Discussion

In the current scenario of urgent demand for alternative chemical control strategies against NWS, this study reports, for the first time, the efficacy of orally administered fluralaner for the prevention of umbilical myiasis in newborn calves. Additionally, it describes unexpected findings regarding the performance of pour-on fluralaner and provides field-based evidence supporting the suspected lack of efficacy of doramectin under practical conditions.

One limitation of this study was that the level of infestation observed in the negative control group was lower than that previously reported for animals of the same category in Brazil [8]. The complete absence of rainfall during the clinical trial, although it was conducted during the rainy season, may have contributed to this finding, as a previous study demonstrated that weekly rainfall patterns are positively associated with the prevalence of umbilical myiasis, with a greater number of rainy days leading to a higher number of affected calves [8]. Nevertheless, the proportion of 26% of infested animals in the control group was sufficient to allow for statistical analysis and to achieve the objectives of the present study.

Regarding the safety pharmacology of fluralaner in newborn calves, although the commercial product containing 5% fluralaner for pour-on use has received conditional approval by the FDA for the prevention and treatment of myiasis caused by *C. hominivorax* in beef cattle 2 months of age and older, and in Brazil, the product label states that clinical safety has not been established in animals younger than 4 months of age, a previous study conducted to assess the potential effect of treating young animals on the enzootic stability of bovine tick fever showed that 50 calves treated at 25 days of age exhibited no adverse effects [11]. Consistently, in the present study, none of the 100 animals treated with fluralaner, either orally or as a pour-on formulation, showed any adverse events.

The results of this experiment indicate that, under the evaluated conditions, the 1% doramectin-based antiparasitic did not meet the expected criteria for the prevention of neonatal umbilical myiasis. These findings suggest the emergence of a doramectin-resistant population of *C. hominivorax*. Studies conducted in Brazil during the 1990s demonstrated 100% prophylactic efficacy of doramectin against bovine myiasis [6, 12, 13]. Although reduced efficacy has become evident under field conditions, to date only two scientific reports have confirmed the lack of efficacy of doramectin against NWS myiasis. In Brazil, injectable ivermectin and doramectin showed preventive efficacies of only 13.3% and 33.3%, respectively, against umbilical myiasis in newborn beef calves [8]. More concerningly, in Argentina, Muchiut et al. [3] reported that 1% doramectin and 3.15% ivermectin formulations showed no detectable efficacy in preventing myiasis caused by *C. hominivorax* in castrated calves. Beyond the issue of reduced efficacy, safety concerns have also been reported. On a farm where the primary justification for the use of avermectins was the prevention of umbilical myiasis in newborn calves, two outbreaks of doramectin poisoning occurred following an accidental overdose of the drug. After a clinical course lasting 24–48 h, a total of 91 calves died [9]. However, in that case, the calves were treated with a 3.5% doramectin formulation, whereas a 1% formulation was used in the present study. Although no direct comparative studies are available, differences in concentration, as well as in the formulation vehicles, may influence the pharmacokinetic profile, and therefore could potentially have implications for clinical safety.

Although 5% fluralaner demonstrated high preventive efficacy, the relative risk reduction (RRR) value below 100% indicates that the product did not fully meet the regulatory criterion under the specific conditions of this study. According to Ordinance No. 48/1997 of the Brazilian Ministry of Agriculture, Livestock and Food Supply (MAPA), systemic antiparasitic products intended for the control of myiasis must achieve 100% efficacy within the first 72 h after treatment, a parameter conceptually equivalent to obtaining 100% relative risk reduction during this initial period.

Despite the product being identical in dose and route of administration to that evaluated in four previous clinical studies in which 100% curative and preventive efficacy was reported [4], a particular factor was observed in the animal category assessed here. Some newborn calves were licked at the application site by their dams shortly after treatment, as illustrated in an additional video file (see Additional File 1). In treated calves that were not licked, the characteristic green coloration of the product remained visible on the skin, whereas this coloration was no longer perceptible in licked animals (see Additional File 2).

This effect appeared to be associated with the occurrence of umbilical myiasis, as exclusively the two calves that were licked at the application site developed myiasis, suggesting that partial removal of the product may have compromised its preventive efficacy. Although the potential effect of rainfall immediately after treatment, which could hypothetically wash the product off the skin, remains unknown, no rainfall occurred within 24 h after treatment of any animal receiving the pour-on formulation in the present study. Future studies based on field efficacy trials combined with pharmacokinetic and pharmacodynamic analyses, such as those performed by Muchiut et al. [3], may help elucidate whether licking of the calf’s skin by the dam or rainfall occurring shortly after pour-on treatment reduces fluralaner concentrations at the lesion site or in the umbilical region.

Nevertheless, the preventive effect observed with 5% fluralaner was substantial and clinically relevant, suggesting its potential application as a complementary tool in neonatal management, particularly in situations where complete risk elimination is not feasible, especially in the current scenario of reduced preventive efficacy of avermectins.

In this study, an innovative 1% oral fluralaner formulation was evaluated and achieved 100% relative risk reduction for the occurrence of umbilical myiasis in treated animals. This route of administration may be highly advantageous for the prevention of myiasis due to its rapid absorption. Although no studies have been conducted in calves, studies in dogs have shown that fluralaner is readily absorbed after single-dose oral administration, reaching maximum plasma concentrations within 1 day [14]. Importantly, newborn calves are not yet functionally ruminant, which supports a cautious extrapolation of these pharmacokinetic findings from monogastric species such as dogs. An additional finding supporting the preventive efficacy of this treatment was that all treated calves presented umbilical lesion scores of zero. These results may provide a scientific basis for preventive programs in endemic areas, particularly in newborn calves, as well as in regions experiencing the reemergence of New World screwworm (NWS), and even in areas currently free of this fly but potentially at risk of reintroduction.

## Conclusions

The findings demonstrate the lack of efficacy of doramectin in preventing neonatal umbilical myiasis, reinforcing concerns regarding parasite resistance to avermectins under field conditions. In contrast, fluralaner showed high preventive efficacy. However, the route of administration directly influenced treatment efficacy, indicating that this factor should be carefully considered when establishing prophylactic protocols for the control of umbilical myiasis in calves.

## Supplementary Information

Supplementary Material 1.

Supplementary Material 2.

## Data Availability

All data supporting the conclusions of this study are available in the article and its Supplementary Information.
